# A comparison of cardiac motion analysis software packages: application to left ventricular deformation analysis in hypertensive patients

**DOI:** 10.1186/1532-429X-17-S1-P57

**Published:** 2015-02-03

**Authors:** Haifa M Almutairi, Filip Zemrak, Thomas A Treibel, Daniel Sado, Redha Boubertakh, Marc E Miquel, Steffen E Petersen

**Affiliations:** 1 William Harvey Research institution, Queen Marry University of London, London, UK; 2 Queen Marry University of London, London, UK; 3 Barts Health NHS Hospital Trust, London, London, UK; 4 Cardiovascular Biomedical Research Unit, Barts and the London School of Medicine and Dentistry, Queen Mary, University of London, London, UK; 5 The Heart Hospital Imaging Centre, University College London, London, UK

## Background

Although myocardial function is clinically assessed with global measurements (ventricular volumes, ejection fraction), recent research has shown that regional measurements, such as wall-thickening, strain, and torsion, could provide earlier sub-clinical markers to examine left ventricular (LV) dysfunction and myocardial diseases.

Cardiovascular Magnetic Resonance myocardial feature tracking (CMR-FT) technique is used to post-process cine CMR images to provide a quantitative assessment of LV motion deformation parameters. It derives myocardial motion deformation from image features such as myocardium-blood cavity boundary and pixel intensities, and relies only on standard cine images to extract motion deformation. The main objective of this study is to compare two current feature tracking software packages in hypertensive patients.

## Methods

29 hypertensive subjects were prospectively recruited from a tertiary hypertension clinic and enrolled to undergo CMR examinations. All images were acquired using a 1.5T scanner (Siemens Medical Imaging, Germany), and a cardiac surface coil. LV function was assessed with cine acquisitions in the following planes: 2-chamber, 4-chamber and short-axis slices (basal, mid and apical levels).

LV deformation was analysed using: 2D Cardiac Performance Analysis, MR (TomTec Imaging Systems, Munich, Germany) and CVI42 (Circle Cardiovascular Imaging Inc. Calgary, Canada). Endocardial and epicardial LV contours were drawn manually at the end diastolic phase in order to achieve best tracking results; the software packages then allow semi-automated analysis to provide quantitative measurement of global and regional deformation parameters.

## Results

Results of circumferential, radial, and longitudinal strains are given in table 1. Statistical analysis was performed using the student's paired t-test for dependent sample in order to assess the difference between the two software packages.All radial strain mean values obtained with CVI42 were higher than with Tomtec and the difference was statistically significant for all short-axis circumferential strains. This was also the case for short-axis apical and 4-chamber radial strains. In total five parameters (short-axis apical and 4-chamber radial strains, all 3 short-axis slices circumferential strains) were statistically different and 5 were not.

**Table 1:** Summary of FT parameters obtained by CVI42 and TomTec. Data are presented as mean±standard deviation (SD). (*) Shows a statistically significant p-value (<0.05).

	Parameters	TomTec Mean±SD	CVI42 Mean±SD	p-value
Radial Strain (%)	Short-axis basal	34.98±9.78	37.71±9.85	0.21

Radial Strain (%)	Short-axis mid	32.65±13.04	35.72±13.37	0.34

Radial Strain (%)	Short-axis apical	25.96±13.83	49.85±24.19	<0.001*

Radial Strain (%)	2-Chamber	32.11±13.33	36.84±9.03	0.11

Radial Strain (%)	4-Chamber	26.27±8.14	38.32±15.38	<0.001*

Circumferential Strain (%)	Short-axis basal	−25.79±5.11	−19.38±3.58	<0.001*

Circumferential Strain (%)	Short- axis mid	−24.93±5.79	−18.3±4.43	<0.001*

Circumferential Strain (%)	Short-axis apical	−29.05±7.03	−22.14±6.45	<0.001*

Longitudinal Strain (%)	2-Chamber	−22.21±6.89	−17.62±3.04	0.025

Longitudinal Strain (%)	4-Chamber	−21.52±7.18	−19.87±10.75	0.45

## Conclusions

From our results, there is a trend in circumferential strain in short-axis (apical, mid, and basal) where there is a significant difference in the values obtained by the two software packages, whereas radial and longitudinal strain values showed no clear trend. Therefore, there is a clear need for a gold standard validation to assess the accuracy of cardiac motion analysis software packages.

## Funding

This project is funded by a Saudi scholarship to the author.

